# Metagenomic analysis of the honey bee queen microbiome reveals low bacterial diversity and Caudoviricetes phages

**DOI:** 10.1128/msystems.01182-23

**Published:** 2024-01-23

**Authors:** Lílian Caesar, Danny W. Rice, Alison McAfee, Robyn Underwood, Carrie Ganote, David R. Tarpy, Leonard J. Foster, Irene L. G. Newton

**Affiliations:** 1 Department of Biology, Indiana University, Bloomington, Indiana, USA; 2 Department of Biochemistry and Molecular Biology, Michael Smith Laboratories, University of British Columbia, Vancouver, British Columbia, Canada; 3 Department of Applied Ecology, North Carolina State University, Raleigh, North Carolina, USA; 4 Department of Entomology, Pennsylvania State University, University Park, State College, Pennsylvania, USA; 5 Luddy School of Informatics, Indiana University, Bloomington, Indiana, USA; University of Connecticut, Storrs, Connecticut, USA

**Keywords:** symbiosis, bacteriophage, eusocial insect, genetic background, *Apis mellifera*

## Abstract

In eusocial insects, the health of the queens—the colony founders and sole reproductive females—is a primary determinant for colony success. Queen failure in the honey bee *Apis mellifera*, for example, is a major concern of beekeepers who annually suffer colony losses, necessitating a greater knowledge of queen health. Several studies on the microbiome of honey bees have characterized its diversity and shown its importance for the health of worker bees, the female non-reproductive caste. However, the microbiome of workers differs from that of queens, which, in comparison, is still poorly studied. Thus, direct investigations of the queen microbiome are required to understand colony-level microbiome assembly, functional roles, and evolution. Here, we used metagenomics to comprehensively characterize the honey bee queen microbiome. Comparing samples from different geographic locations and breeder sources, we show that the microbiome of queens is mostly shaped by the environment experienced since early life and is predicted to play roles in the breakdown of the diet and protection from pathogens and xenobiotics. We also reveal that the microbiome of queens comprises only four candidate core bacterial species, *Apilactobacillus kunkeei*, *Lactobacillus apis, Bombella apis*, and *Commensalibacter* sp. Interestingly, in addition to bacteria, we show that bacteriophages infect the queen microbiome, for which Lactobacillaceae are predicted to be the main reservoirs. Together, our results provide the basis to understand the honey bee colony microbiome assemblage, can guide improvements in queen-rearing processes, and highlight the importance of considering bacteriophages for queen microbiome health and microbiome homeostasis in eusocial insects.

**IMPORTANCE:**

The queen caste plays a central role in colony success in eusocial insects, as queens lay eggs and regulate colony behavior and development. Queen failure can cause colonies to collapse, which is one of the major concerns of beekeepers. Thus, understanding the biology behind the queen’s health is a pressing issue. Previous studies have shown that the bee microbiome plays an important role in worker bee health, but little is known about the queen microbiome and its function *in vivo*. Here, we characterized the queen microbiome, identifying for the first time the present species and their putative functions. We show that the queen microbiome has predicted nutritional and protective roles in queen association and comprises only four consistently present bacterial species. Additionally, we bring to attention the spread of phages in the queen microbiome, which increased in abundance in failing queens and may impact the fate of the colony.

## INTRODUCTION

Queen health in eusocial insects is a primary determinant for the success of the colony. Eusocial insects live in groups with cooperative care of juveniles, overlap of generations, and reproductive division of labor. Exemplars of insect societies are ants, wasps, and bees from the order Hymenoptera, the largest and most well-known animal group with eusocial species. In eusocial insects, the reproductive female caste (queens) is responsible for laying eggs and regulating the colony’s behavior and development. After mating, queens spend almost their entire life, which can reach several decades, inside the colony being fed by specialized workers. They are among the most fecund terrestrial animals; queens from some insect species can lay ~20,000 eggs per day (1), which may explain the success of some eusocial insect genera. The importance of queens also extends beyond their reproductive role. Queens maintain the colony homeostasis by managing the behavior of other colony members, such as attracting workers or inducing submissive behavior, modulating aggression, or inhibiting the production of new queens through pheromone signals (2–6).

The importance of queens for colony success becomes even more evident with the example of honey bees (*Apis mellifera*). In addition to the significance of honey bees for general ecosystem services in their natural range, managed colonies contribute billions to the agricultural economy annually in the United States, due to their pollination services (7). However, annual losses of honey bee-managed colonies have reduced reliability on them and affected food security. Queen failure and premature supersedure are consistently reported as the leading contributing factors for colony mortality (8). Honey bee queens can live 3–4 years, but recently, their diminished longevity requires the replacement of the queens almost every year, a practice also used preventively (9). The causes behind failing queens are still poorly understood, but they may include problems with development, insemination success, infection by parasites, exposure to xenobiotics, and adverse temperatures (10, 11). Interestingly, the microbiome of insects, including honey bee foragers, is also known to modulate the host response to some of these stressors. For example, the worker gut microbiome can protect the host from parasites (12), and parasites can also shape the gut microbiome due to the infection (13); or the microbiome can buffer the effect of pesticides (14), and pesticides can shape the microbiomes (15), ultimately impacting colony fitness. Thus, the microbiome is an important trait that should be included in studies on the health of eusocial insect queens, and *Apis mellifera* is an excellent model system for these investigations.

Most of what is known about the microbiome of honey bees and its role in colony health comes from investigations on worker bees—the non-reproductive females that take care of the hive and when older leave the colony to collect pollen and nectar. Honey bee workers have a taxonomically simple and consistent gut microbiome among colonies around the world, comprising the core members *Bifidobacterium*, *Snodgrassella*, *Gilliamella*, and two groups of *Lactobacillus,* Firm-4 and Firm-5. This microbial community impacts host health in multiple ways (see reference 16), and its acquisition and transmission occur mostly through interactions with individuals from the colony and the hive environment (17). Dysbiosis in this system is strongly correlated with poorer worker fitness and is characterized by shifts in the load of core microbes and the spread of opportunistic bacteria (18). More recently, fungi and bacteriophages’ community characterization has also been included in studies of the bee microbiome (19–23). The role that phages play in molding the microbiome and impacting host health has been shown in unrelated model systems (24–27), but in honey bees, their diversity and potential effects are understudied.

Curiously, there is evidence that honey bee queen microbes are distinct from those of workers from their own colony (28). Thus far, based on 16S rRNA amplicon-based studies, the honey bee queen microbiome appears to comprise mostly bacteria from the genera *Lactobacillus*, *Bombella,* and *Commensalibacter*. However, the queen gut microbiome is not as consistent as observed for worker bees, and the relative abundance of the associated bacteria is quite variable among queens from different colonies (28–30). In addition to the natural microbiome variability among queens, controlled experiments have shown that both age and early-bacteria colonizers coming from social interactions or rearing protocols also lead to differences in their microbiomes (29). A metagenomic approach, enabling species-level characterization and access to genomic information, could improve our understanding of the queen microbiome assemblage and its role in queen health.

Here, we sequenced the metagenomes of 18 queen gut samples from the United States and Canada to characterize their microbiome. These queen samples have associated metadata from previous studies (31, 32), including both failing and healthy queens. We describe the queen gut microbial community, from bacteria to phages, and investigate the most important factors shaping it. In addition, we characterize the microbiome at a functional level and recover metagenome-assembled genomes to identify, at the species level, the candidate core microbiome.

## RESULTS

### The honey bee queen microbiome is dominated by bacteria of the Acetobacteraceae and Lactobacillaceae families

**Fig 1:**
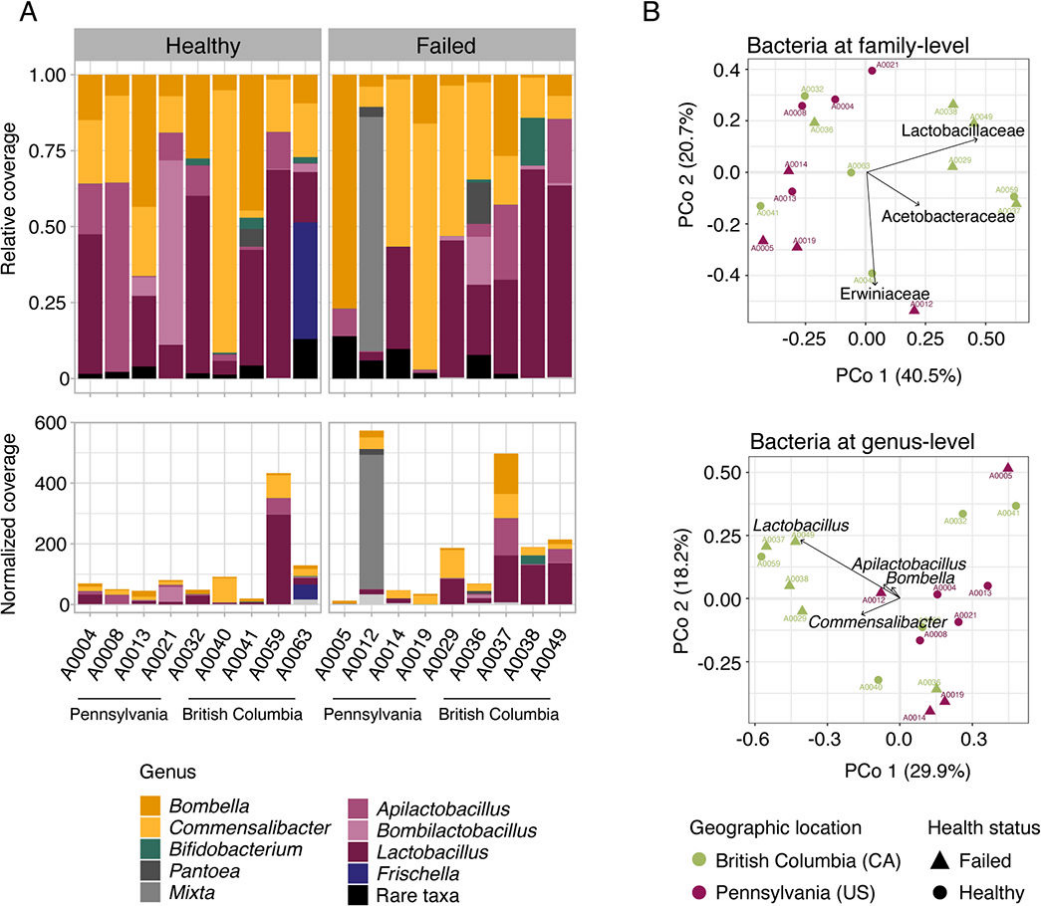
The microbiome of honey bee queens varies extensively in the abundance of the two most prevalent bacterial families, Acetobacteraceae and Lactobacillaceae. (A) Relative (above) and library read depth normalized (below) coverage of bacteria genera comprising the microbiome of healthy and failing honey bee queens from two different locations, Pennsylvania (USA) and British Columbia (Canada). Color shades represent bacteria families; Acetobacteraceae (yellow), Bifidobacteriaceae (green), Erwiniaceae (gray), Lactobacillaceae (purple), and Orbaceae (blue). (B) Principal coordinate analysis (PCoA) showing the distribution of queen samples based on the dissimilarity of relative proportions of the microbial communities at family (above) and genus level (below). Arrows highlight the bacteria with the strongest contribution to the ordination, and each axis shows the percentage of variation explaining.

We sequenced a total of 18 queen gut metagenomes, ranging from 7 to 33 million paired reads per sample after quality trimming (Table S1). First, we asked which microbes were present in the community associated with the honey bee queen gut. We mapped trimmed reads against 18S rRNA and 16S rRNA databases for taxonomic identification of fungi and bacteria, respectively. As expected, based on the paucity of fungal reads recovered from other studies, no reads mapped to 18S rRNA; on the other hand, reads mapped to bacteria from at least five families, mostly Acetobacteraceae and Lactobacillaceae (Fig. 1A). At the genus level, the queen microbiomes varied extensively in the abundance of Acetobacteraceae (*Bombella* or *Commensalibacter*) and Lactobacillaceae (*Apilactobacillus* or *Lactobacillus*) (Fig. 1A). Other bacteria, known to often be a part of the worker bee gut microbiome, were also present in some of the queen samples, such as *Bombilactobacillus*, *Bifidobacterium,* and *Frischella*. The geographical location from which queens were sampled (PERMANOVA; City, bacterial family, *P* = 0.046, *R*^2^ = 0.304, *F* = 2.165; bacterial genus *P* = 0.045, *R*^2^ = 0.252, *F* = 1.667; State, bacterial family, *P* = 0.047, *R*^2^ = 0.115, *F* = 2.270; bacterial genus, *P* = 0.039, *R*^2^ = 0.121, *F* = 2.286) and the year of sampling (which is conflated with the geographic location, see Table S1) were two of the factors explaining the differences between the queen microbiomes both at the family and genus level. Additionally, the queen source—that is, the breeder who provided the queen—was also a factor explaining microbiome differences across samples (PERMANOVA; bacterial family, *P* = 0.040, *R*^2^ = 0.421, *F* = 2.488, bacterial genus, *P* = 0.039, *R*^2^ = 0.357, *F* = 1.850). Queen source, although influenced by the genetics of the stocks, could also be influenced by environmental differences since the queen-rearing environment and protocol used by the beekeeper could impact queen microbiomes. Importantly, in our study, the location of these samples (State and City) is also confounded by the queen source, as beekeepers from the same area generally had the same queen suppliers (Table S1).

Due to the effect of the environment on the queen microbiome composition, differences in the microbiome of healthy or failing queens were only observed depending on their geographical location (PERMANOVA; interaction between State and Status, *P* = 0.047, *R*^2^ = 0.121, *F* = 2.395). This interaction effect was observed at the family level; the queen core microbiome families, Lactobacillaceae and Acetobacteraceae, were among the taxa that explain most of these differences among the samples and their clustering in the principal coordinate analysis, PCoA (Fig. 1B). But also, Erwiniaceae, a non-core bacterial family of the honey bee microbiome, was more abundant in failing queens and contributed to the PCoA topology separating sample A0012 from the other U.S. samples (Fig. 1B). Interestingly, with the normalization of the bacteria coverage by the library read depth, we can also observe that failing queens, mostly from British Columbia (Canada), have higher proportions of bacterial reads per gut sample (Fig. 1A). This result, however, could also be affected by differences in diet consumption (environmental DNA) on the day of sampling, and future studies could use quantitative PCR to obtain absolute bacterial abundances.

### Queen genetic background is not directly correlated with microbiome composition or health status

**Fig 2:**
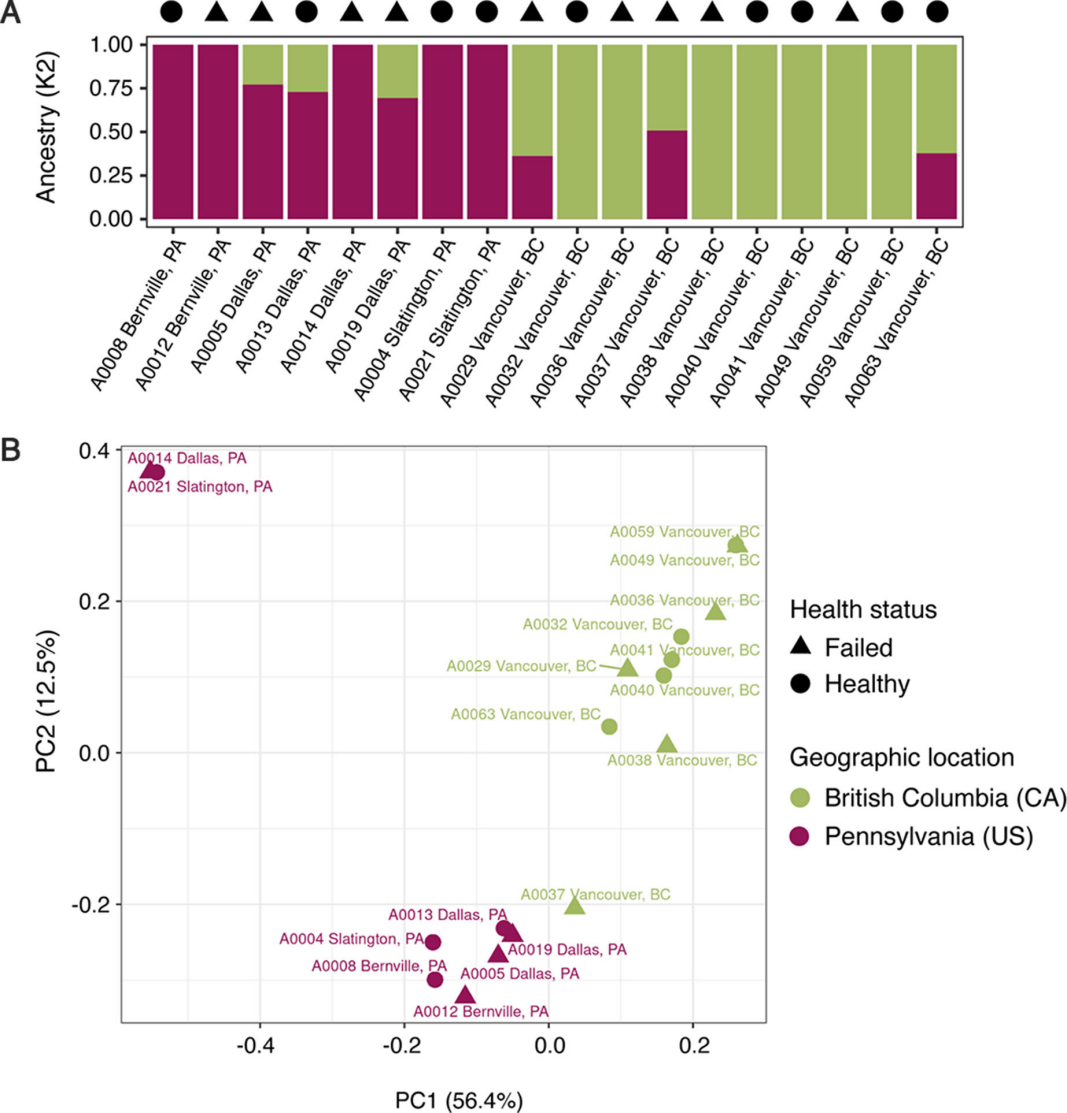
Genetic background of the honey bee queens groups them in a population for each general geographical location and not by health status or microbiome composition. (A) Bar plot resulting from the SNP admixture analysis for K2 ancestral populations (*y*-axis shows the proportion of each ancestral population), clustering queens from Pennsylvania (PA) and British Columbia (BC). (B) PCA of the genotype likelihood covariance matrix shows the same population structure, separated in PC1, which explains 22% of the total variation.

Because the queen source—that is, the breeder who provided the queen—was one of the factors explaining microbiome composition, we decided to further investigate if the queen’s genetic background could be a direct predictor of its gut community. Honey bee queen population structure was assessed from genotype likelihoods of 7,342,540 polymorphic sites. Both population structure plots showed a clear genetic differentiation among the honey bee queens from Pennsylvania (USA) and British Columbia (Canada); although the admixture plot suggests some gene flow between populations (Fig. 2A). We also tested higher numbers of ancestral populations and there is no clear population structure for queens from the same apiary or queen company supplier (Fig. S1). The principal component analysis (PCA) also cleanly delineates the samples based on the State factor and interestingly, the samples with mixed ancestry are those at the edges of each cluster, closer to the center of PC1 (Fig. 2B). Finally, we used a Mantel test to compare similarity/dissimilarity of queen genetic background and microbiome composition. The bacterial families or genera were not correlated with pairwise genetic distance among queens (bacterial family, *R* = 0.04113, *P* = 0.3906; bacterial genus *R* = 0.1676, *P* = 0.1152) or covariance matrix of queen genotype likelihoods (bacterial family, *R* = 0.05412, *P* = 0.2925; bacterial genus *R* = −0.04525, *P* = 0.727).

### The candidate core microbiome of queens comprises four bacterial species

**Fig 3:**
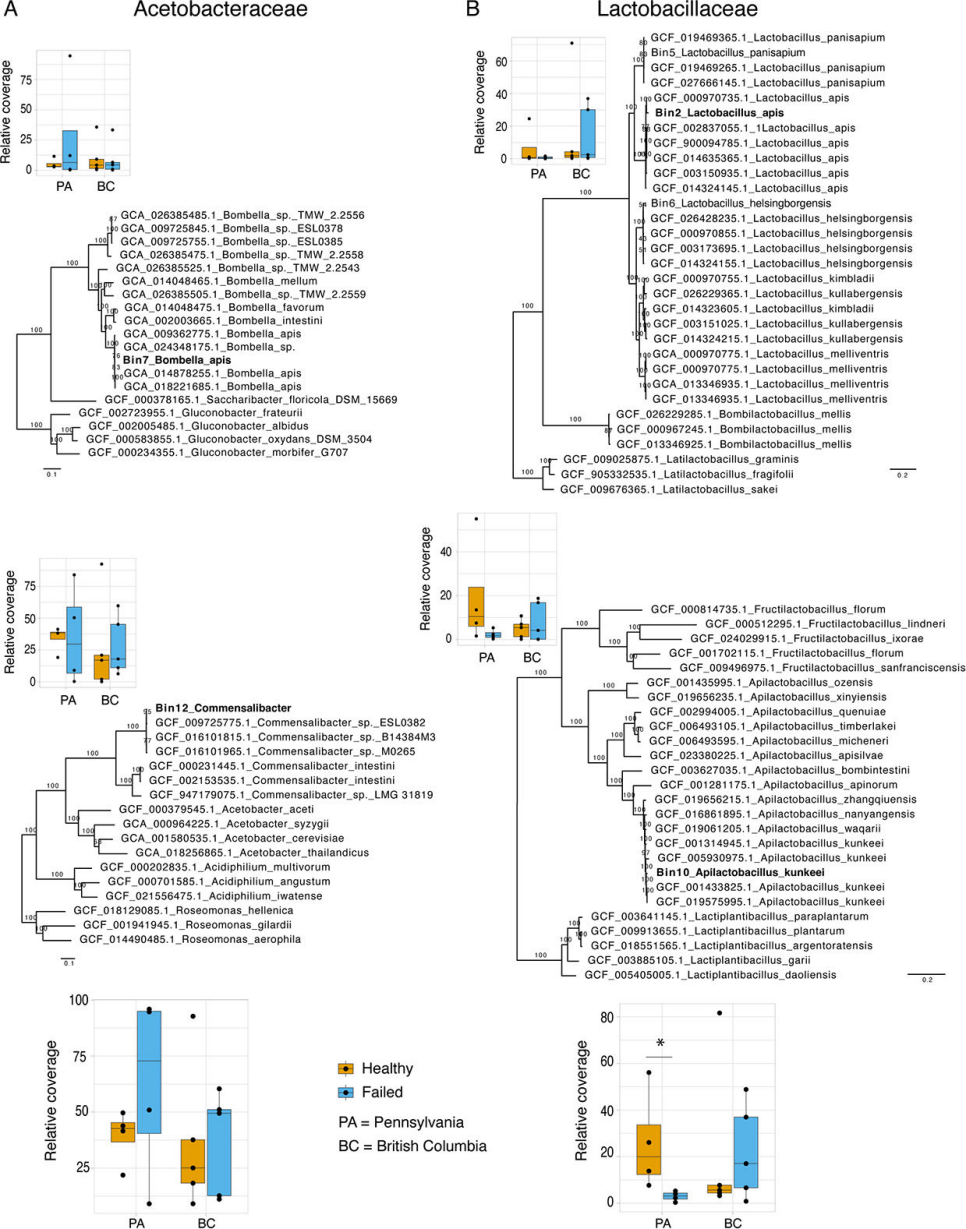
The core microbiome of honey bee queens comprises only four species. (A) Phylogenies inferred with maximum likelihood for the MAGs of the two core Acetobacteraceae. Above, *Bombella apis* and relatives, inferred from the alignment of 927 single-copy orthologs (SCO). Below, *Commensalibacter* and relatives, inferred from the alignment of 708 SCO. (B) Phylogenies for the MAGs of the two core Lactobacillaceae. Above, *Lactobacillus apis* and relatives*,* inferred from the alignment of 80 SCO. Below, *Apilactobacillus kunkeei* and relatives*,* inferred from the alignment of 292 SCO. (A and B) On the side of each phylogeny is the relative coverage of the MAG in queens with different health statuses and geographical locations. Below the phylogenies of each bacterial family is the same plot configuration, but with the coverage sum of both MAGs. Significant statistical difference was only observed when comparing the abundance of core Lactobacillaceae among queens with different health statuses in Pennsylvania; there is a reduction of their abundance in failing queens (Wilcoxon rank test, *P = 0.028).

We recovered metagenome-assembled genomes (MAGs) of the core honey bee queen microbiome members. The co-assembly of all trimmed reads generated 177,846 contigs > 500 nt (Table S2), which were then grouped into bins. In total, eight bacterial MAGs with partial (<50%) to nearly complete (>90%) genomes were recovered and placed into a phylogenetic context to predict species (Table S3; Fig. 3; Fig. S2). Considering that MAGs will be retrieved from the abundant species in metagenomes (present in more samples, with more reads), among them we should be able to find the core members of the queen microbiome. Core microbiome members should be present in all healthy queens and some of the failing queens, while non-core members would be present only in a few queen samples. Single-copy core genes were used to estimate the coverage of MAGs, revealing that *Bombella apis*, *Commensalibacter* sp., *Apilactobacillus kunkeei,* and *Lactobacillus apis* were present in all queen samples (with the exception of *L. apis,* which was absent in one failing queen; Fig. 3; Table S4). In 11 out of 18 samples, these four bacteria species represent more than 50% of the microbiome (Table S4). Only the sample A0012, a failing queen, had an important reduction of the core microbiome (9%) and an increase in the abundance of Enterobacteriaceae. This opportunistic bacterium is part of the non-core MAGs, which includes the Lactobacillaceae *Lactobacillus panisapium*, the Bifidobacteriaceae *Bifidobacterium apousia*, the Orbaceae *Frischella perrara*, and the Enterobacteriaceae *Tenebrionicola*-like (Fig. S2).

We also used these MAGs to ask if a shift in the microbial community, i.e., dysbiosis, was observed in the abundance of the core species from the microbiome of failing queens. Although we can observe a tendency for a shift in species proportions when comparing queens with different health statuses, variance of relative coverage within the group was high, and there was no significant difference and no shift pattern in their proportions in failing queens across both locations (Fig. 3). At the family level, however, *Lactobacillus* in the state of Pennsylvania had a significant decrease in their proportion in failing queens (Wilcoxon rank test, *P* = 0.028).

### Functional properties of the queen gut microbiome

**Fig 4:**
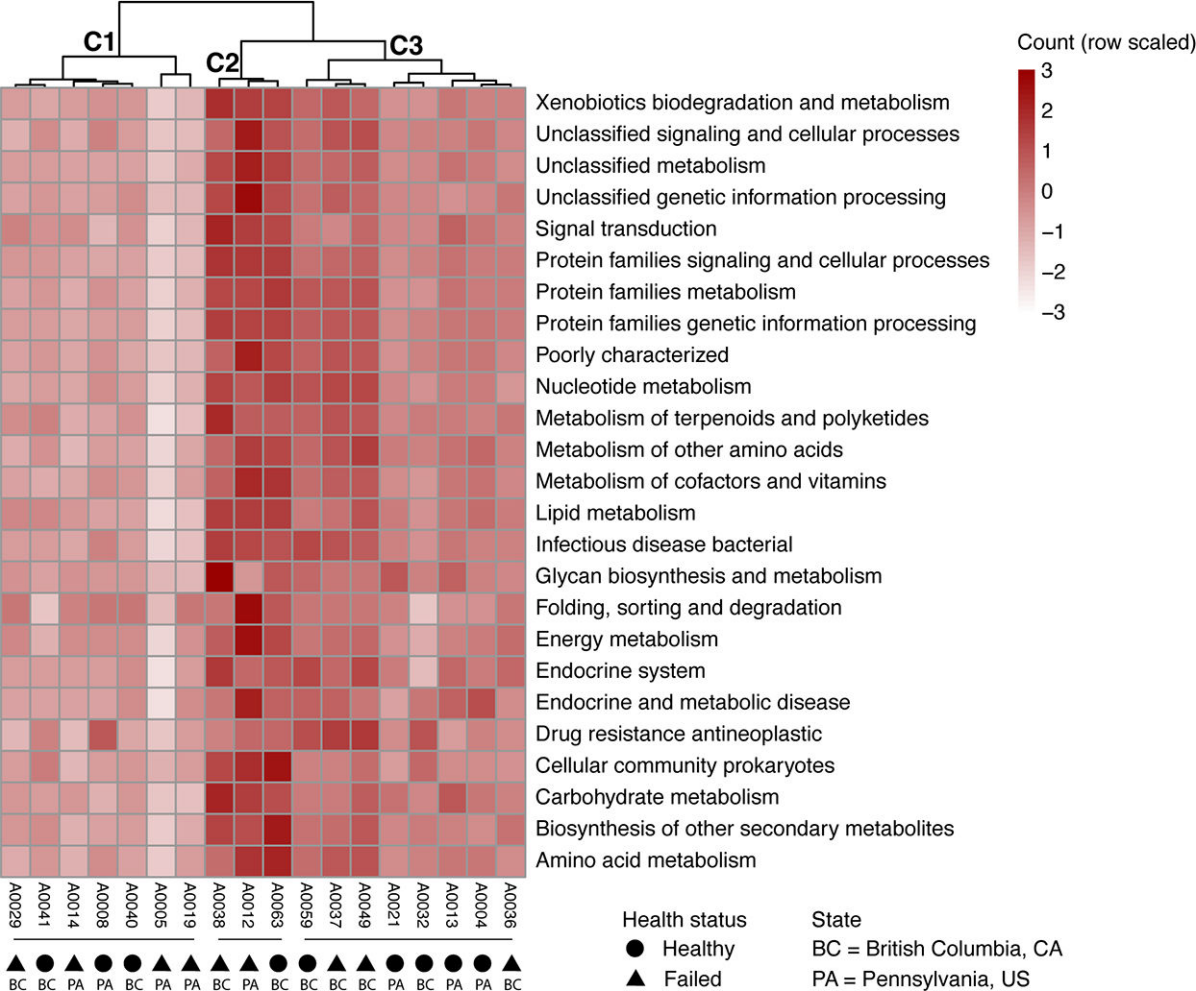
The microbiome functional profile suggests an important role for honey bee queen health and homeostasis. The heatmap shows scaled protein counts per KEGG category (rows), which include proteins involved in xenobiotics biodegradation and metabolism, amino acid metabolism biosynthesis (e.g., lysine), or biosynthesis of secondary metabolites (e.g., antibiotics). Samples (columns) were hierarchically clustered. Three main functional groups were formed (C1, C2, and C3), but they do not correlate with any of the variables tested in our investigations, such as health status or geographic location. Cluster 2, however, has an increase (dark red) in the representation of all KEGG categories due to the relative abundance of non-core bacteria in their microbiomes.

To characterize the potential role of the microbiome in the queen host and interactions with and within the microbial community, we predicted protein functions from all bacterial contigs—thus avoiding biases due to MAG completion status or non-binned partial bacteria genomes, which may also have proteins with functional relevance. From all bacterial contigs, 23,833 proteins were predicted and 12,910 (54%) were assigned to Kyoto Encyclopedia of Genes and Genomes (KEGG) ortholog categories. The presence and absence of proteins in each sample were used to compare them in a clustered heatmap, where all functional categories in the queen microbiome are shown (Fig. 4). Overall, there was no broad functional category missing or overrepresented in samples, so no clustering by microbiome functional profile was observed for samples of the same geographic location (Pennsylvania vs British Columbia) or health status. Cluster 2 (C2, Fig. 4), however, includes the samples with more abundant non-core bacteria. In addition to proteins with general cell function, the main categories present were the metabolism of cofactors and vitamins, energy metabolism, carbohydrate metabolism, and amino acid metabolism. Protein counts from the energy metabolism category confirm that the microbes associated with honey bee queens mostly use oxidative phosphorylation for energy production (Fig. S3E). These microbes also harbor genes involved in the degradation of xenobiotics (e.g., cytochrome P450), the biosynthesis of antibiotics (e.g., monobactam and streptomycin), and biofilm formation pathways, which may play an important role in community regulation (Fig. S3M, B, and D, respectively). While genes for lysine biosynthesis were annotated in all samples, only sample A0012, a failing queen with depleted core-microbiome and increased abundance of the Enterobacteriaceae *Tenebrionicola*-like, has an increase in proteins involved in the degradation of this amino acid, known to be able to buffer poor bee nutrition (Fig. S3A) (33).

### Bacteriophages of the class Caudoviricetes compose the queen microbiome and are found in higher abundance in failing queens

In addition to the search for fungi and bacteria in the microbiome of queens, we also wondered if bacteriophages, important modulators of microbial communities, could also compose the microbiome of the honey bee queens. A total of 74 phage viral operational taxonomic units (vOTUs) were retrieved, including three temperate and 71 putative virulent phages (Table S5). All the phage sequences with predicted genes were classified taxonomically as Caudoviricetes based on the similarity with viral marker genes in the database. Among the temperate phages, one is integrated into the genome of *Lactobacillus panisapium*, and the other two are integrated into the genome of the Enterobacteriaceae, *Tenebrionicola*-like. We tested whether viruses integrated into their host genomes were being induced by comparing the coverage of temperate phages and the coverage of the respective host (Table S6). The coverage of these phages, however, was not greater than the coverage of the genome of their hosts, indicating that they are not being induced.

**Fig 5:**
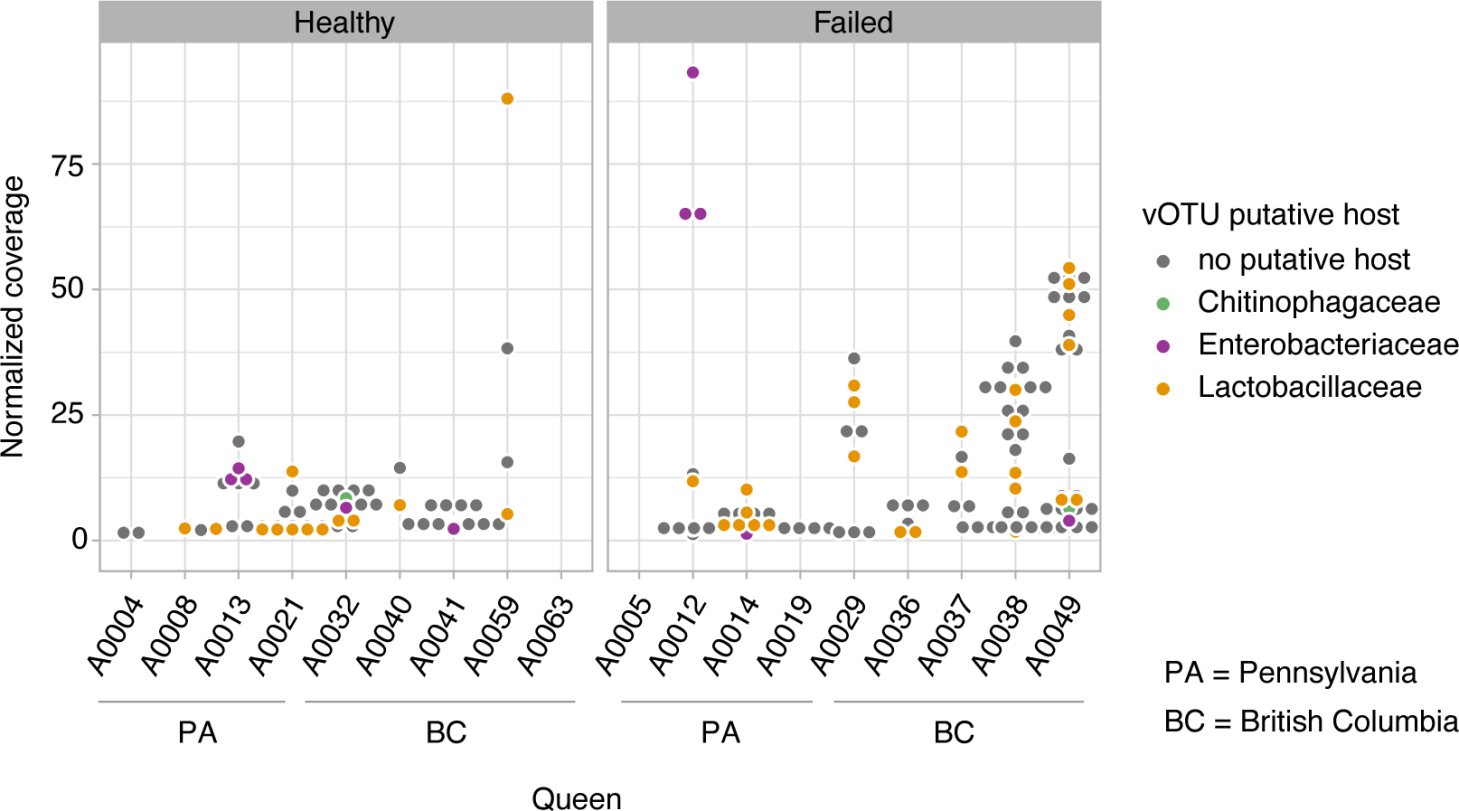
Caudoviricetes phages spread opportunistically in the microbiome of failing queens. The coverage of phage vOTUs (dots, with a coverage >1) is shown for each queen sample, which was higher in failing queens (Wilcoxon rank test, *P* = 0.0297; PA, *P* = 0.032; BC, *P* = 5.12e−5). The color of the dot indicates the host where its genome is integrated or its putative host.

To predict putative hosts for the virulent phage vOTUs, we first conducted nucleotide similarity searches against CRISPR-cas arrays predicted in our MAGs. Genomes of *B. apis, L. apis, L. panisapium,* and *Tenebrionicola*-like have CRISPR-cas arrays with one to seven spacers per array (List S1), but none of the virulent phage sequences from the metagenomes matched these spacers. However, we still were able to predict putative hosts of 21 virulent phages based on the other approaches used, including matches with other CRISPR spacers’ databases, BLASTs, and k-mer compositional analyses (Fig. 5; Table S5). The virulent phages were predicted to infect mostly Lactobacillaceae and Enterobacteriaceae.

Differences in the abundance of phage sequences among the samples were significant for all factors being tested, including geographic location (Kruskal-Wallis rank test; *P* = 0.005, df = 3), age (Wilcoxon rank test; *P* = 8.41e−02), or queen source (Kruskal-Wallis rank test; *P* = 0.005, df = 4). Interestingly, the queen’s health status was one of the most significant factors in terms of differences in phage abundance (Wilcoxon rank test; *P* = 0.029; British Columbia, *P* = 5.12e−05, Pennsylvania, *P* = 0.032), even when excluding temperate phages (Wilcoxon rank test; *P* = 0.04).

Additionally, we estimated the ratio between bacteria and phages in the samples, showing that although queens tend to have more bacteria than phages, the four samples with more phages than bacteria were failed queens (Table S7). Also, since phages need a host, we could expect a consistently increased abundance of phages in the samples with an increased abundance of their hosts. Following this rationale, we used a correlation analysis to potentially find putative hosts for phages with no putative host identified with the previous methods. As expected, all significant correlations among phages and bacteria were positive correlations, and most bacteria co-occurred precisely with phages predicted to infect their family, such as Lactobacillaceae and Enterobacteriaceae. The result of this analysis indicates some phages that may be candidates to infect three members of the core microbiome, *B. apis* (e.g., k141_169038), *A. kunkeei* (e.g., k141_368347), and *Commensalibacter* (e.g., k141_456794; Fig. S4), although these bacteria also had significant, but weaker correlations with Lactobacillaceae-putative phages.

## DISCUSSION

Investigations on the microbiome of eusocial insect queens are not as frequent as those on worker castes, despite the key role of the queen for superorganism fitness. However, studies conducted so far have revealed that the microbiome of queens—including species of termites, ants, and bees—usually differs from that of their workers (28, 34, 35). The lack of direct correlation between worker and queen microbiomes reinforces the importance of queen microbiome characterization to understand colony-level microbiome assembly, functional roles, and evolution. Honey bees, especially, are economically relevant and have been important models for microbiome investigations; amplicon-based characterizations of the queen microbiome have shown that it is mostly composed of Lactobacillaceae and Acetobacteraceae (28–30). Here, using a metagenomic approach, we showed that the microbiome of honey bee queens (i) varies considerably in the abundance of these two bacterial families, with the environment having an important impact on these differences, (ii) the microbiome comprises four core bacterial species and is predicted to have a protective and nutritional role, and that (iii) temperate and virulent Caudoviricetes phages are part of the microbiome, for which core Lactobacillaceae may be important reservoirs and spreaders.

Previous studies on bee workers and queens have shown that, to some extent, their genetic background influences microbiome composition. In workers, these results were obtained by comparing the microbiome of *A. mellifera* subspecies, isolated from each other and inbred since 1980 (36). The evidence for the effect of queen genetic background on microbiome composition was shown by comparing two species of *Apis* (*A. cerana* and *A. mellifera*), which have their last common ancestor dated to approximately 6 mya (37, 38). In the current study, we did not observe the same trends comparing queen genotypes of the same *A. mellifera* subspecies, meaning the genetic differences among the queens were not enough to explain the differences in the microbiome (Fig. 2). This result, however, does not eliminate the possibility of rare alleles playing a role in the queen microbiome assembly, and a larger sample size with queens from more populations could test this hypothesis. Interestingly, we instead found that the queen microbiome composition was shaped by the rearing environment, i.e., geographic location and queen breeder source. For worker bees, it is known that the environment landscape plays an important role in the microbiome composition (39). The effect of breeder source on queen microbiome composition was also observed in another study with honey bee queens (30) and, together with the lack of correlation between queen genetic background and microbiome composition, suggests a role of priority effects (arrival order and/or timing) on queen microbiome assembly. The effect of the environment on microbiome composition may also have masked differences in the microbiome of healthy and failing queens (Fig. 1). Dysbiosis can shift the microbiome to a different end community composition depending on previous colonizers and the factors causing the shift (18). Thus, to characterize dysbiosis patterns in failing queens, an experimental design controlling for environmental effects would be important in future investigations. Regardless, with our approach, we were able to observe a typical dysbiotic pattern in some of the failing queens, which was an increase in bacteria abundance and an increase in the proportion of non-core microbiome members. However, the changes observed in the microbiomes of the failing queens in our study could be a consequence of weak queens and not the direct cause of queen failure.

Our findings reveal that the microbiome of queens is very constrained, composed of only four candidate core bacterial species: *Bombella apis, Commensalibacter* sp.*, Apilactobacillus kunkeei,* and *Lactobacillus apis* (Fig. 3). This represents even fewer bacterial members than the known simple core microbiome of worker bees, comprising five phylotypes with multiple species (40, 41). Additionally, no fungi were detected in our queen samples, which is in contrast to previously published amplicon sequencing-based investigations with queens and worker bees (19, 20, 42, 43). Importantly, this difference could be due to the fact that amplicon sequencing will detect extremely low abundance templates resulting from the honey bee diet.

Queens are the longest-lived members in a colony, they are larger than workers, and they have well‐developed ovaries, thus their physiology itself already represents a different niche for bacteria to colonize. Additionally, the highly specialized diet of queens throughout their lives, comprising mainly royal jelly, may have facilitated the selection of a small number of core members. Queens are genetically identical to their sisters that became workers, i.e., female larvae are bipotent and can equally develop into queens or workers depending on their larval diet (royal jelly vs worker brood food). Royal jelly is composed of water, proteins, sugars, and lipids, but it is also very viscous, acidic, and imbibed with antimicrobial peptides, such as royalisin, jelleines, apismin, royalactin, and fatty acids, which together confer the antimicrobial role of the royal jelly (44, 45). For example, royal jelly can inhibit the growth of bee pathogenic bacteria, such as *Melissococcus plutonius* and *Paenibacillus alvei* (46). Interestingly, royal jelly does not inhibit all bacteria; a recent study showed that *Bombella apis*, one of the core members of the queen microbiome, can withstand and even replicate in royal jelly (33). In this same study, however, *Apilactobacillus kunkeei*, did not show the same ability. In other host model systems where the microbiome is also composed of Acetobacteraceae and Lactobacillaceae, such as in *Drosophila*, the colonization of these bacterial families is enabled due to cross-feeding; *Acetobacter pomorum* uses the lactate produced by *Lactobacillus plantarum* to supply amino acids that are essential to *L. plantarum* (47). All the members of the queen microbiome can be isolated and cultivated in the laboratory, and future experiments could test if cross-feeding is behind the presence of these core bacteria in such an antimicrobial environment. It is also important to note that the royal jelly is produced, secreted, and offered to the queen by nurse bees, meaning the selection for these royal-jelly-resistant microbes is likely already occurring in the nurse bee hypopharyngeal glands. Moreover, it is conceivable that queens have different needs with regard to nutrition compared to workers, and thus they may require different nutritional symbionts. In fact, our functional analysis has shown that the microbiome of queens is equipped with genes involved in protein and carbohydrate metabolism (e.g., peptidases, lipid biosynthesis, fructose, and mannose metabolism; see Fig. 4; Fig. S3), likely supplementing primary components of the host’s diet.

With our metagenomic approach, we were able to show for the first time that Caudoviricetes, a class of tailed bacteriophages, are part of the queen gut microbial community (Table S5). Previous studies have shown that Caudoviricetes are the main phages in the microbiome of honey bee workers (21–23), but the lack of similarity in the gut bacterial community of these two female castes has left open the question about the phages present in the queen microbiome. In the worker’s gut, the main phage hosts are the core bacteria *Bifidobacterium*, *Gilliamella,* and *Lactobacillus,* and the non-core *Bartonella* (21–23). Among these hosts, *Lactobacillus* is the only genus that is also found in the queen microbiome and, interestingly, we show here it is the queen gut bacterium hosting the majority of phages (Fig. 5). Importantly, however, for many phages, the host prediction is difficult to impossible (21–23); here, 77% of the phage sequences did not have a host predicted. However, we indeed predicted CRISPR-Cas systems with spacers in the *Bombella apis* MAG, suggesting a history of infections by phages (List S1). Also, phage sequence distribution was consistent with the change in the microbiome due to environmental effects, and phages were also more abundant in the failing queens (Fig. 5; Table S7). This result points out that dysbiosis, usually detected via shifts in bacterial composition, can also be characterized by phage production and spread in the microbiome. We observed that some of the most abundant phage sequences in the queen microbiome are associated with a non-core bacterium, an Enterobacteriaceae, putting the native queen microbiome at risk. One of these most abundant phages is a temperate phage, based on the analysis of coverage compared to the host genome. If phages are induced or spread in the microbiome, they can not only directly kill their hosts but may also trigger immunological responses or even foster gene transfer between cells (48). The inherent complexity of a eusocial insect colony poses a challenge in investigations on the health of queens and, ultimately, of the colony (49). For future studies, a longitudinal approach could improve the resolution of the ecological interaction feedback between phages and bacteria over time and its impact on queen health or failure.

## MATERIALS AND METHODS

### Honey bee queen samples

Honey bee queens were sampled in 2018 from colonies located in Pennsylvania, USA, and in 2019 from colonies in British Columbia, Canada. The samples from both locations are part of previously published data sets (31, 32), in which the queens were classified according to their health status. Queens were scored as healthy when they showed no sign of supersedure cells, no drone brood in worker cells, no signs of disease, and had strong worker populations. Failing queens exhibited one or more of the following: drone brood in worker cells, spotty brood pattern, weak colony population, and supersedure cells. In addition to the health status, these samples have associated metadata that were used for the analysis, including queen age, source, ovary mass and size, and management strategy (Table S1). All samples were stored at −70°C until dissection and further use.

### Sample processing and shotgun sequencing

All queens were dissected on ice with 70% ethanol sterilized tools by gently pulling on the last abdominal tergite. DNA extraction was performed by a microbiome analytics company, Microbiome Insights, using the Qiagen MagAttract PowerSoil DNA KF Kit optimized for the Thermo Scientific KingFisher robot. Quality and quantity of DNA were checked on an Agilent 2200 TapeStation, and a total of 18 samples with a DNA integrity number > 6 were submitted for library construction and metagenome sequencing at the biotechnology company SeqCenter, Pittsburgh, PA, USA. Libraries were prepared using the Illumina DNA Prep kit and IDT 10 bp UDI indices and sequenced on an Illumina NextSeq 2000, producing 2 × 151-bp reads. Demultiplexing, quality control, and adapter trimming were performed with bcl-convert (v3.9.3) from Illumina. Sequencing data can be found within the NCBI BioProject PRJNA1007366.

### Bacteria and fungi community characterization

Paired-end raw sequencing reads were first trimmed by length and quality using Trimmomatic v.0.36, with options “LEADING:28 TRAILING:28 SLIDINGWINDOW:6:25 MINLEN:75” (50). Bowtie2 v.2.4.2 was used to map trimmed individual sample reads to bacteria and fungi marker gene databases, using the options “--no-discordant --very-sensitive” (51). The bacteria sequence database used was BEExact, a comprehensive 16S rRNA database of all bacteria taxa previously found associated with bees (52). The fungal sequence database used was SILVA 138.1 SSU, which contains 18S rRNA sequences (53). Samtools was used to transform mapping files and retrieve, with depth command, option “-a,” the coverage per base of the marker gene sequence (54). Coverage was summed for sequences from the same family or genus and normalized by the sequence length and library read depth (mean sequence coverage/normalization factor; Table S1).

### Co-assembly and broad taxonomic assignment

Trimmed reads from all 18 samples were used in a co-assembly with MEGAHIT v.1.1.2 (55). Contigs > 500 nt were binned into metagenome-assembled genomes (see the section Metagenome-assembled bacteria genomes) and used for broad taxonomic classification of samples. We first classified bee contigs by mapping a 500 nt random fragment of contigs with Bowtie2 v.2.4.2 against *Apis mellifera* genome, GCF_003254395.2_Amel_HAv3.1 (51). Contigs that did not map were used as queries in a BLASTx (56) against nr database from the NCBI, being classified according to the best hit taxonomy as bacteria, eukaryote/bee, virus, or unknown (Table S2). Contig coverage used for the following analyses was recovered by mapping trimmed individual sample reads back to all contigs with Bowtie2 v.2.4.2, using the options “--no-discordant --very-sensitive” (51). Samtools was used for file transformation, and the depth command with option “-a” was used to recover the coverage per base of the contigs (54). Mapping results were also used to plot the proportion of reads mapping to host or other taxa (Fig. S5).

### Metagenome-assembled genomes

Contigs were grouped into bins with MetaBAT2 v.2.11.3 using default settings (57). CheckM v.1.1.6 was used to assess the quality of the resulting MAGs (58). Bacterial MAGs with >30% completeness and <5% contamination were included in the following analyses. To confirm that dereplication of MAGs is not necessary, which is expected for co-assemblies, we ran the “dereplicate” command from dRep v.3.4.2 (59). No MAGs were clustered beyond the threshold of >90% average nucleotide identity from the primary dendrogram of pair-wise Mash distances between all MAGs. Each MAG was subjected to gene/protein prediction with Prokka v.1.14.6, using default options (60). In addition, we used CRISPRCasFinder with default parameters to identify CRISPR-Cas loci in contigs from all MAGs (61). CRISPR repeat spacers from all CRISPR arrays were extracted and added to a spacer database used for putative phage host discovery (see “Characterization of bacteriophages in the microbiome”; List S1).

### Phylogenetic placement of MAGs and abundance

MAGs were placed into phylogenetic trees with related genomes for species-level taxonomic characterization. The best BLASTx results of the MAG contigs (see “Co-assembly and broad taxonomic assignment”) guided the choice of species reference genomes to retrieve from NCBI and include in the phylogenetic analysis, in addition to outgroups. Single-copy orthologs were recovered with OrthoFinder v.2.5.4 (62), aligned with MAFFT v.7.520 (63), and then concatenated for IQTREE v.2.2.0.3 maximum likelihood analysis, options -m TEST -B 1000 (64). Figtree v.1.4.4 was used for tree visualization. To estimate and compare MAG abundance across samples, the mean coverage of single-copy orthologs was retrieved from previous mapping output (see “Co-assembly and broad taxonomic assignment”) with an in-house Perl script (https://github.com/liliancaesarbio/general_scripts/) and normalized to the library read depth (mean sequence coverage/normalization factor; Table S1). The proportion of total reads mapping to each MAG was also recovered and made available in Table S8.

### Microbiome functional characterization

All contigs classified taxonomically as bacteria, including MAG sequences, had proteins predicted with Prokka using default settings (60). The mean coverage of each protein was retrieved from previous mapping (see “Co-assembly and broad taxonomic assignment”) with an in-house Perl script (https://github.com/liliancaesarbio/general_scripts/). Proteins were assigned for functionality at different levels of the KEGG database with EggNOG-mapper 2.1, using default parameters (65). Genes with >0.1× coverage were considered present. First, KEGG level B of description was retried for each KO ID, and categories with total samples sum of >700 coverage and 100 protein counts were considered. Gene counts for both KEGG level B and KEGG level C for each non-general function category were plotted with R v.3.6.3, using package pheatmap (66). To test for functional similarity between the samples, the heatmap was hierarchically clustered with hclust complete linkage method.

### Characterization of bacteriophages in the microbiome

Sequences of bacteriophages were identified using three programs; geNomad was run with option “end-to-end” and a cutoff of >0.7 virus score (67), VirSorter2 v2.2.4 was run using options “--min-score 0.7 --hallmark-required-on-short” (68), and VIBRANT v1.2.1 was run using the default settings (69). For this analysis, contigs > 500 nt, classified as bacteria, virus, or unknown, were used as input, enabling the identification of virulent or temperate phages. For contigs of bacteria, only proviruses (temperate phages) were considered, since the other detected phage sequences may represent horizontal gene transfer events. No viral bins were generated or dereplication needed due to the co-assembly approach (see Metagenome-assembled genomes”); also, no bins were recovered with vRhyme v1.1.0, using default settings (70). CheckV “end-to-end” was used to estimate completeness of the final list of viral operational taxonomic units (71), and only phage vOTUs with at least one non-host gene were retained for the following analyses. The mean coverage of vOTUs was recovered from the depth output files previously obtained (see “Co-assembly and broad taxonomic assignment”) using an in-house script (https://github.com/liliancaesarbio/general_scripts/). Coverage was estimated based only on the viral region of the contig since they are the result of a co-assembly, and in some samples that virus may not be present—as for the case of temperate phages. Candidate hosts were predicted with iPHoP v.1.3.1 (72), BLASTn with options “-evalue 1e-3 -ungapped -perc_identity 95” against NCBI nt database, CRISPR spaces annotated from MAGs and also against the CrisprOpenDB (73). The last database includes more than 9 billion spacers, as well as the ones annotated in previously published bee microbiome studies (21–23).

### Queen genetic background

Trimmed reads from individual samples were mapped against *Apis mellifera* reference genome (GCF_003254395.2_Amel_HAv3.1) with Bowtie2 v.2.4.2, using the option “--no-discordant --very-sensitive” (51). Bam files were used to generate the beagle file of genotype likelihoods with ANGSD, options “-GL 2 -doGlf 2 -doMajorMinor 1 -SNP_pval 1e-6 -doMaf 1” (74). Since microbiome metagenome sequencing does not aim at a great sequencing depth of the host genome, here we follow the recommended approach of using genotype likelihoods to circumvent the uncertainty of host genotypes due to low or medium host sequencing coverage (75). Genetic structure and individual queen ancestry proportions were analyzed with NGSadmix, option -minMaf 0.05, for clusters (K) ranging from 2 to 5 (76). Population structure was further investigated by partitioning the genetic variance using a principal component analysis based on genotype likelihoods using PCAngsd (77). The contribution of each principal component was calculated in R and plotted using ggplot2 (78). As an alternative approach, used later for correlation analysis with microbiome composition, we also estimated pairwise genetic distances directly, using genotype likelihoods, with ngsDist (79). The input file for ngsDist was prepared with ANGSD, options “-minMapQ 20 -minQ 20 -doCounts 1 -Gl 1 -doMajorMinor 1 -doMaf 1 -SNP_pval 1e-6 -doGeno 8 -doPost 1.”

### Statistical analysis

All graphs and analyses were run on R v.3.6.3 using the packages cited. We assessed beta diversity and clustering profiles for the 16S rRNA-based results with a PCoA ordination plot ran on Bray-Curtis dissimilarity matrices using package Ape (80), then plotted with ggplot2 (78). To test for the effects of geographic location (State and City), year, age, queen origin, management, health status, and fitness markers (ovary size and ovary mass) on the bacterial community composition, we ran PERMANOVA, 9,999 permutations, with the function adonis2 from Vegan package (81). The effect of the same factors on the proportion of core microbiome members and Caudoviricetes was tested with *t*-test or Wilcoxon rank test and Kruskal-Wallis rank test in the case data were not normally distributed with Shapiro-Wilk normality test. All *P*-values were adjusted with the false discovery rate method. To test for the co-occurrence of phages and bacteria, we ran a Spearman correlation analysis with package Hmisc (82), plotted with packages corrplot (83) and PerformanceAnalytics (84). To test for the correlation between host genetic background and microbiome composition, we used the Mantel test from package Vegan (81), with Spearman correlation and 9,999 permutations. The input matrices were the queen genotype likelihood covariance matrix or the queen pairwise genetic distance (see “Queen genetic background”) and the Bray-Curtis dissimilarity of the gut microbiome composition at the family or genus level.

## Data Availability

Sequencing data can be found within the NCBI BioProject PRJNA1007366.

## SUPPLEMENTAL MATERIAL

List S1 msystems.01182-23-s0001.fasta Listed spacers from microbiome members.

Figure S1 msystems.01182-23-s0002.tif Admixture analysis of sampled queen populations.

Figure S2 msystems.01182-23-s0003.tif Phylogenies of non-core bacteria present in honey bee queens.

Figure S3 msystems.01182-23-s0004.tif Zoom-in on non-general function KEGG categories.

Figure S4 msystems.01182-23-s0005.tif Correlation between MAG size and phage abundance.

Figure S5 msystems.01182-23-s0006.tif Proportion of reads mapping to host and microbes.

Tables S1 to S8 msystems.01182-23-s0007.xlsx Supplementary tables of MAG contamination, completeness, and other details.

ASM does not own the copyrights to Supplemental Material that may be linked to, or accessed through, an article. The authors have granted ASM a non-exclusive, world-wide license to publish the Supplemental Material files. Please contact the corresponding author directly for reuse.

## References

[1] Sieber R, Leuthold RH. 1982. Development and physiogastry in the queen of the fungus-growing termite Macrotermes michaelseni (Isoptera: Macrothermitnae). J Insect Physiol 28:979–985. doi:10.1016/0022-1910(82)90002-6

[2] Slessor KN, Kaminski LA, King GGS, Borden JH, Winston ML. 1988. Semiochemical basis of the retinue response to queen honey bees. Nature 332:354–356. doi:10.1038/332354a0

[3] Vargo EL. 1988. A bioassay for a primer pheromone of queen fire ants (Solenopsis invicta) which inhibits the production of sexuals. Ins Soc 35:382–392. doi:10.1007/BF02225813

[4] Winston ML, Higo HA, Slessor KN. 1990. Effect of various dosages of queen mandibular gland pheromone on the inhibition of queen rearing in the honey bee (Hymenoptera: Apidae). Ann Entomol Soc Am 83:234–238. doi:10.1093/aesa/83.2.234

[5] Smith AA, Hölldober B, Liebig J. 2009. Cuticular hydrocarbons reliably identify cheaters and allow enforcement of altruism in a social insect. Curr Biol 19:78–81. doi:10.1016/j.cub.2008.11.059 19135369

[6] Smith AA, Millar JG, Suarez AV. 2016. Comparative analysis of fertility signals and sex-specific cuticular chemical profiles of Odontomachus trap-jaw ants. J Exp Biol 219:419–430. doi:10.1242/jeb.128850 26847561

[7] Khalifa SAM, Elshafiey EH, Shetaia AA, El-Wahed AAA, Algethami AF, Musharraf SG, AlAjmi MF, Zhao C, Masry SHD, Abdel-Daim MM, Halabi MF, Kai G, Al Naggar Y, Bishr M, Diab MAM, El-Seedi HR. 2021. Overview of bee pollination and its economic value for crop production. Insect 12:688. doi:10.3390/insects12080688 PMC8396518 34442255

[8] National Agricultural Statistics Service (NASS). 2021. Issn, p 1949–1492. In Agricultural Statistics board, United States Department of Agriculture (USDA). https://downloads.usda.library.cornell.edu/usda-esmis/files/hd76s004z/7h14bh90x/w9505v43v/hony0321.pdf.

[9] Rangel J, Keller JJ, Tarpy DR. 2013. The effects of honey bee (Apis mellifera L.) queen reproductive potential on colony growth. Ins Soc 60:65–73. doi:10.1007/s00040-012-0267-1

[10] Amiri E, Strand MK, Rueppell O, Tarpy DR. 2017. Queen quality and the impact of honey bee diseases on queen health: potential for interactions between two major threats to colony health. Insect 8:48. doi:10.3390/insects8020048 PMC5492062 28481294

[11] McAfee A, Tarpy DR, Foster LJ. 2021. Queen honey bees exhibit variable resilience to temperature stress. PLoS One 16:e0255381. doi:10.1371/journal.pone.0255381 34379669 PMC8357134

[12] Schwarz RS, Moran NA, Evans JD. 2016. Early gut colonizers shape parasite susceptibility and microbiota composition in honey bee workers. Proc Natl Acad Sci USA 113:9345–9350. doi:10.1073/pnas.1606631113 27482088 PMC4995961

[13] Paris L, Peghaire E, Moné A, Diogon M, Debroas D, Delbac F, El Alaoui H. 2020. Honeybee gut microbiota dysbiosis in pesticide/parasite co-exposures is mainly induced by Nosema ceranae. J Invertebr Pathol 172:107348. doi:10.1016/j.jip.2020.107348 32119953

[14] Almasri H, Liberti J, Brunet JL, Engel P, Belzunces LP. 2022. Mild chronic exposure to pesticides alters physiological markers of honey bee health without perturbing the core gut microbiota. Sci Rep 12:4281. doi:10.1038/s41598-022-08009-2 35277551 PMC8917129

[15] Motta EVS, Raymann K, Moran NA. 2018. Glyphosate perturbs the gut microbiota of honey bees. Proc Natl Acad Sci U S A 115:10305–10310. doi:10.1073/pnas.1803880115 30249635 PMC6187125

[16] Raymann K, Moran NA. 2018. The role of the gut microbiome in health and disease of adult honey bee workers. Curr Opin Insect Sci 26:97–104. doi:10.1016/j.cois.2018.02.012 29764668 PMC6010230

[17] Miller DL, Parish AJ, Newton IL. 2019. Transitions and transmission: behavior and physiology as drivers of honey bee-associated microbial communities. Curr Opin Microbiol 50:1–7. doi:10.1016/j.mib.2019.08.001 31563000

[18] Anderson KE, Ricigliano VA. 2017. Honey bee gut dysbiosis: a novel context of disease ecology. Curr Opin Insect Sci 22:125–132. doi:10.1016/j.cois.2017.05.020 28805634

[19] Yun JH, Jung MJ, Kim PS, Bae JW. 2018. Social status shapes the bacterial and fungal gut communities of the honey bee. Sci Rep 8:2019. doi:10.1038/s41598-018-19860-7 29386588 PMC5792453

[20] Callegari M, Crotti E, Fusi M, Marasco R, Gonella E, De Noni I, Romano D, Borin S, Tsiamis G, Cherif A, Alma A, Daffonchio D. 2021. Compartmentalization of bacterial and fungal microbiomes in the gut of adult honeybees. NPJ Bio Microbio 7:42. doi:10.1038/s41522-021-00212-9 PMC8105395 33963194

[21] Bonilla-Rosso G, Steiner T, Wichmann F, Bexkens E, Engel P. 2020. Honey bees harbor a diverse gut virome engaging in nested strain-level interactions with the microbiota. Proc Natl Acad Sci USA 117:7355–7362. doi:10.1073/pnas.2000228117 32179689 PMC7132132

[22] Deboutte W, Beller L, Yinda CK, Maes P, de Graaf DC, Matthijnssens J. 2020. Honey-bee-associated prokaryotic viral communities reveal wide viral diversity and a profound metabolic coding potential. Proc Natl Acad Sci USA 117:10511–10519. doi:10.1073/pnas.1921859117 32341166 PMC7229680

[23] Busby TJ, Miller CR, Moran NA, Van Leuven JT. 2022. Global composition of the bacteriophage community in honey bees. mSystems 7:e0119521. doi:10.1128/msystems.01195-21 35343797 PMC9040601

[24] Oh JH, Lin XB, Zhang S, Tollenaar SL, Özçam M, Dunphy C, Walter J, van Pijkeren JP. 2019. Prophages in Lactobacillus reuteri are associated with fitness trade-offs but can increase competitiveness in the gut ecosystem. Appl Environ Microbiol 86:e01922-19. doi:10.1128/AEM.01922-19 31676478 PMC6912086

[25] Džunková M, Low SJ, Daly JN, Deng L, Rinke C, Hugenholtz P. 2019. Defining the human gut host-phage network through single-cell viral tagging. Nat Microbiol 4:2192–2203. doi:10.1038/s41564-019-0526-2 31384000

[26] Heyer R, Schallert K, Siewert C, Kohrs F, Greve J, Maus I, Klang J, Klocke M, Heiermann M, Hoffmann M, Püttker S, Calusinska M, Zoun R, Saake G, Benndorf D, Reichl U. 2019. Metaproteome analysis reveals that syntrophy, competition, and phage-host interaction shape microbial communities in biogas plants. Microbiome 7:69. doi:10.1186/s40168-019-0673-y 31029164 PMC6486700

[27] Dragoš A, Andersen AJC, Lozano-Andrade CN, Kempen PJ, Kovács ÁT, Strube ML. 2021. Phages carry interbacterial weapons encoded by biosynthetic gene clusters. Curr Biol 31:3479–3489. doi:10.1016/j.cub.2021.05.046 34186025

[28] Tarpy DR, Mattila HR, Newton ILG. 2015. Development of the honey bee gut microbiome throughout the queen-rearing process. Appl Environ Microbiol 81:3182–3191. doi:10.1128/AEM.00307-15 25724964 PMC4393441

[29] Powell JE, Eiri D, Moran NA, Rangel J. 2018. Modulation of the honey bee queen microbiota: effects of early social contact. PLoS One 13:e0200527. doi:10.1371/journal.pone.0200527 30001407 PMC6042773

[30] Copeland DC, Anderson KE, Mott BM. 2022. Early queen development in honey bees: social context and queen breeder source affect gut microbiota and associated metabolism. Microbiol Spectr 10:e0038322. doi:10.1128/spectrum.00383-22 35867384 PMC9430896

[31] McAfee A, Chapman A, Higo H, Underwood R, Milone J, Foster LJ, Guarna MM, Tarpy DR, Pettis JS. 2020. Vulnerability of honey bee queens to heat-induced loss of fertility. Nat Sustain 3:367–376. doi:10.1038/s41893-020-0493-x

[32] McAfee A, Chapman A, Pettis JS, Foster LJ, Tarpy DR. 2021. Trade-offs between sperm viability and immune protein expression in honey bee queens (Apis mellifera). Commun Biol 4:48. doi:10.1038/s42003-020-01586-w 33420325 PMC7794525

[33] Parish AJ, Rice DW, Tanquary VM, Tennessen JM, Newton ILG. 2022. Honey bee symbiont buffers larvae against nutritional stress and supplements lysine. ISME J 16:2160–2168. doi:10.1038/s41396-022-01268-x 35726020 PMC9381588

[34] Hu Y, D’Amelio CLD, Béchade B, Cabuslay CS, Sanders JG, Price S, Fanwick E, Powell S, Moreau CS, Russell JA. 2021. The secrets to domestic bliss – partner fidelity and environmental filtering preserve stage-specific turtle ant gut symbioses for over 40 million years. bioRxiv. doi:10.1101/2021.09.13.460005

[35] Poulsen M, Hu H, Li C, Chen Z, Xu L, Otani S, Nygaard S, Nobre T, Klaubauf S, Schindler PM, Hauser F, Pan H, Yang Z, Sonnenberg ASM, de Beer ZW, Zhang Y, Wingfield MJ, Grimmelikhuijzen CJP, de Vries RP, Korb J, Aanen DK, Wang J, Boomsma JJ, Zhang G. 2014. Complementary symbiont contributions to plant decomposition in a fungus-farming termite. Proc Natl Acad Sci USA 111:14500–14505. doi:10.1073/pnas.1319718111 25246537 PMC4209977

[36] Wu J, Lang H, Mu X, Zhang Z, Su Q, Hu X, Zheng H. 2021. Honey bee genetics shape the strain-level structure of gut microbiota in social transmission. Microbiome 9:225. doi:10.1186/s40168-021-01174-y 34784973 PMC8597283

[37] Chen C, Wang H, Liu Z, Chen X, Tang J, Meng F, Shi W. 2018. Population genomics provide insights into the evolution and adaptation of the eastern honey bee (Apis cerana). Mol Biol Evol 35:2260–2271. doi:10.1093/molbev/msy130 29931308 PMC6107058

[38] Yang J, Zhong Y, Xu L, Zeng B, Lai K, Yang M, Li D, Zhao Y, Zhang M, Li D. 2021. The dominating role of genetic background in shaping gut microbiota of honeybee queen over environmental factors. Front Microbiol 12:722901. doi:10.3389/fmicb.2021.722901 34803942 PMC8603915

[39] Jones JC, Fruciano C, Hildebrand F, Al Toufalilia H, Balfour NJ, Bork P, Engel P, Ratnieks FL, Hughes WO. 2018. Gut microbiota composition is associated with environmental landscape in honey bees. Ecol Evol 8:441–451. doi:10.1002/ece3.3597 29321884 PMC5756847

[40] Kwong WK, Medina LA, Koch H, Sing K-W, Soh EJY, Ascher JS, Jaffé R, Moran NA. 2017. Dynamic microbiome evolution in social bees. Sci Adv 3:e1600513. doi:10.1126/sciadv.1600513 28435856 PMC5371421

[41] Ellegaard KM, Engel P. 2019. Genomic diversity landscape of the honey bee gut microbiota. Nat Commun 10:446. doi:10.1038/s41467-019-08303-0 30683856 PMC6347622

[42] Anderson KE, Ricigliano VA, Copeland DC, Mott BM, Maes P. 2023. Social interaction is unnecessary for hindgut microbiome transmission in honey bees: the effect of diet and social exposure on tissue-specific microbiome assembly. Microb Ecol 85:1498–1513. doi:10.1007/s00248-022-02025-5 35499645 PMC10167169

[43] Decker LE, San Juan PA, Warren ML, Duckworth CE, Gao C, Fukami T. 2023. Higher variability in fungi compared to bacteria in the foraging honey bee gut. Microb Ecol 85:330–334. doi:10.1007/s00248-021-01922-5 34997310

[44] Alreshoodi FM, Sultanbawa Y. 2015. Antimicrobial activity of royal jelly. 1 st. Vol. 13. Bentham Science Publishers, Sharjah, United Arab Emirates.

[45] Fratini F, Cilia G, Mancini S, Felicioli A. 2016. Royal jelly: an ancient remedy with remarkable antibacterial properties. Microbiol Res 192:130–141. doi:10.1016/j.micres.2016.06.007 27664731

[46] Vezeteu TV, Bobiş O, Moritz RFA, Buttstedt A. 2017. Food to some, poison to others - honeybee royal jelly and its growth inhibiting effect on European foulbrood bacteria. Microbiologyopen 6:e00397. doi:10.1002/mbo3.397 27743422 PMC5300887

[47] Henriques SF, Dhakan DB, Serra L, Francisco AP, Carvalho-Santos Z, Baltazar C, Elias AP, Anjos M, Zhang T, Maddocks ODK, Ribeiro C. 2020. Metabolic cross-feeding in imbalanced diets allows gut microbes to improve reproduction and alter host behaviour. Nat Commun 11:4236. doi:10.1038/s41467-020-18049-9 32843654 PMC7447780

[48] Brown TL, Charity OJ, Adriaenssens EM. 2022. Ecological and functional roles of bacteriophages in contrasting environments: marine, terrestrial and human gut. Curr Opin Microbiol 70:102229. doi:10.1016/j.mib.2022.102229 36347213

[49] López-Uribe MM, Ricigliano VA, Simone-Finstrom M. 2020. Defining pollinator health: a holistic approach based on ecological, genetic, and physiological factors. Annu Rev Anim Biosci 8:269–294. doi:10.1146/annurev-animal-020518-115045 31618045

[50] Bolger AM, Lohse M, Usadel B. 2014. Trimmomatic: a flexible trimmer for illumina sequence data. Bioinform 30:2114–2120. doi:10.1093/bioinformatics/btu170 PMC4103590 24695404

[51] Langmead B, Salzberg SL. 2012. Fast gapped-read alignment with bowtie 2. Nat Methods 9:357–359. doi:10.1038/nmeth.1923 22388286 PMC3322381

[52] Daisley BA, Reid G. 2021. BEExact: a metataxonomic database tool for high-resolution inference of bee-associated microbial communities. mSystems 6:e00082-21. doi:10.1128/mSystems.00082-21 33824193 PMC8546966

[53] Quast C, Pruesse E, Yilmaz P, Gerken J, Schweer T, Yarza P, Peplies J, Glöckner FO. 2013. The SILVA ribosomal RNA gene database project: improved data processing and web-based tools. Nucleic Acids Res 41:D590–6. doi:10.1093/nar/gks1219 23193283 PMC3531112

[54] Li H, Handsaker B, Wysoker A, Fennell T, Ruan J, 1000 Genome Project Data Processing Subgroup. 2009. The sequence alignment/map format and samtools. BMC Bioinformatics 25:2078–2079. doi:10.1093/bioinformatics/btp352 PMC2723002 19505943

[55] Li D, Liu C-M, Luo R, Sadakane K, Lam T-W. 2015. MEGAHIT: an ultra-fast single-node solution for large and complex metagenomics assembly via succinct de bruijn graph. Bioinform 31:1674–1676. doi:10.1093/bioinformatics/btv033 25609793

[56] Camacho C, Coulouris G, Avagyan V, Ma N, Papadopoulos J, Bealer K, Madden TL. 2009. BLAST+: architecture and applications. BMC Bioinform 10:421. doi:10.1186/1471-2105-10-421 PMC2803857 20003500

[57] Kang DD, Li F, Kirton E, Thomas A, Egan R, An H, Wang Z. 2019. MetaBAT 2: an adaptive binning algorithm for robust and efficient genome reconstruction from metagenome assemblies. PeerJ 7:e7359. doi:10.7717/peerj.7359 31388474 PMC6662567

[58] Parks DH, Imelfort M, Skennerton CT, Hugenholtz P, Tyson GW. 2015. CheckM: assessing the quality of microbial genomes recovered from isolates, single cells, and metagenomes. Genome Res 25:1043–1055. doi:10.1101/gr.186072.114 25977477 PMC4484387

[59] Olm MR, Brown CT, Brooks B, Banfield JF. 2017. dRep: a tool for fast and accurate genomic comparisons that enables improved genome recovery from metagenomes through de-replication. ISME J 11:2864–2868. doi:10.1038/ismej.2017.126 28742071 PMC5702732

[60] Seemann T. 2014. Prokka: rapid prokaryotic genome annotation. Bioinform 30:2068–2069. doi:10.1093/bioinformatics/btu153 24642063

[61] Couvin D, Bernheim A, Toffano-Nioche C, Touchon M, Michalik J, Néron B, Rocha EPC, Vergnaud G, Gautheret D, Pourcel C. 2018. CRISPRCasFinder, an update of CRISRFinder, includes a portable version, enhanced performance and integrates search for Cas proteins. Nucleic Acids Res 46:W246–W251. doi:10.1093/nar/gky425 29790974 PMC6030898

[62] Emms DM, Kelly S. 2019. OrthoFinder: phylogenetic orthology inference for comparative genomics. Genome Biol 20:238. doi:10.1186/s13059-019-1832-y 31727128 PMC6857279

[63] Katoh K, Standley DM. 2013. MAFFT multiple sequence alignment software version 7: improvements in performance and usability. Mol Biol Evol 30:772–780. doi:10.1093/molbev/mst010 23329690 PMC3603318

[64] Minh BQ, Schmidt HA, Chernomor O, Schrempf D, Woodhams MD, von Haeseler A, Lanfear R. 2020. IQ-TREE 2: new models and efficient methods for phylogenetic inference in the genomic era. Mol Biol Evol 37:2461. doi:10.1093/molbev/msaa131 32011700 PMC7182206

[65] Cantalapiedra CP, Hernández-Plaza A, Letunic I, Bork P, Huerta-Cepas J. 2021. eggNOG-mapper v2: functional annotation, orthology assignments, and domain prediction at the metagenomic scale. Mol Biol Evol 38:5825–5829. doi:10.1093/molbev/msab293 34597405 PMC8662613

[66] Kolde R. 2019. Pheatmap: pretty heatmaps. CRAN R project. Available from: https://cran.r-project.org/web/packages/pheatmap/index.html

[67] Camargo AP, Roux S, Schulz F, Babinski M, Xu Y, Hu B, Chain PSG, Nayfach S, Kyrpides NC. 2023. Identification of mobile genetic elements with geNomad. Nat Biotechnol 21. doi:10.1038/s41587-023-01953-y PMC11324519 37735266

[68] Guo J, Bolduc B, Zayed AA, Varsani A, Dominguez-Huerta G, Delmont TO, Pratama AA, Gazitúa MC, Vik D, Sullivan MB, Roux S. 2021. VirSorter2: a multi-classifier, expert-guided approach to detect diverse DNA and RNA viruses. Microbiome 9:37. doi:10.1186/s40168-020-00990-y 33522966 PMC7852108

[69] Kieft K, Zhou Z, Anantharaman K. 2020. VIBRANT: automated recovery, annotation and curation of microbial viruses, and evaluation of viral community function from genomic sequences. Microbiome 8:90. doi:10.1186/s40168-020-00867-0 32522236 PMC7288430

[70] Kieft K, Adams A, Salamzade R, Kalan L, Anantharaman K. 2022. vRhyme enables binning of viral genomes from metagenomes. Nucleic Acids Res 50:e83. doi:10.1093/nar/gkac341 35544285 PMC9371927

[71] Nayfach S, Camargo AP, Schulz F, Eloe-Fadrosh E, Roux S, Kyrpides NC. 2021. CheckV assesses the quality and completeness of metagenome-assembled viral genomes. Nat Biotechnol 39:578–585. doi:10.1038/s41587-020-00774-7 33349699 PMC8116208

[72] Roux S, Camargo AP, Coutinho FH, Dabdoub SM, Dutilh BE, Nayfach S, Tritt A, Arumugam M. 2023. iPHoP: an integrated machine learning framework to maximize host prediction for metagenome-derived viruses of archaea and bacteria. PLoS Biol 21:e3002083. doi:10.1371/journal.pbio.3002083 37083735 PMC10155999

[73] Dion MB, Plante PL, Zufferey E, Shah SA, Corbeil J, Moineau S. 2021. Streamlining CRISPR spacer-based bacterial host predictions to decipher the viral dark matter. Nucleic Acids Res 49:3127–3138. doi:10.1093/nar/gkab133 33677572 PMC8034630

[74] Korneliussen TS, Albrechtsen A, Nielsen R. 2014. ANGSD: analysis of next generation sequencing data. BMC Bioinformatics 15. doi:10.1186/s12859-014-0356-4 PMC4248462 25420514

[75] Nielsen R, Paul JS, Albrechtsen A, Song YS. 2011. Genotype and SNP calling from next-generation sequencing data. Nat Rev Genet 12:443–451. doi:10.1038/nrg2986 21587300 PMC3593722

[76] Skotte L, Korneliussen TS, Albrechtsen A. 2013. Estimating individual admixture proportions from next generation sequencing data. Genetics 195:693–702. doi:10.1534/genetics.113.154138 24026093 PMC3813857

[77] Meisner J, Albrechtsen A. 2018. Inferring population structure and admixture proportions in low-depth NGS data. Genetics 210:719–731. doi:10.1534/genetics.118.301336 30131346 PMC6216594

[78] Wickham H. 2016. CRAN R project. ggplot2: elegant graphics for data analysis. Available from: http://link.springer.com/10.1007/978-3-319-24277-4

[79] Vieira FG, Lassalle F, Korneliussen TS, Fumagalli M. 2016. Improving the estimation of genetic distances from next-generation sequencing data. Biol. J. Linn. Soc 117:139–149. doi:10.1111/bij.12511

[80] Paradis E, Schliep K, Schwartz R. 2019. Ape 5.0: an environment for modern phylogenetics and evolutionary analyses in R. CRAN R project 35:526–528. doi:10.1093/bioinformatics/bty633 30016406

[81] Oksanen J, Simpson GL, Blanchet FG, Kindt R, Legendre P, Minchin PR, O’Hara RB, Solymos P, Stevens MHH, Szoecs E, Wagner E, Barbour M, Bedward M, Bolker B, et al.. 2022. Vegan: community ecology package. CRAN R package. doi:10.1021/acs.jpclett.2c01302

[82] Harrell Jr F. 2022. Hmisc: harrell miscellaneous. CRAN R project. https://cran.r-project.org/web/packages/Hmisc/index.html.

[83] Wei T, Simko V. 2021. CRAN R package. Corrplot: visualization of a correlation matrix. Available from: https://cran.r-project.org/web/packages/corrplot/index.html

[84] Peterson BG, Carl P. 2020. CRAN R package. PerformanceAnalytics: econometric tools for performance and risk analysis. Available from: https://cran.r-project.org/web/packages/PerformanceAnalytics/index.html

