# Supplementary figures and images for: Metagenomic analysis of the honey bee queen microbiome reveals low bacterial diversity and Caudoviricetes phages
Source: mSystems. 2024 Jan 23;9(2):e01182-23. doi: 10.1128/msystems.01182-23 (PMC10878037; doi:10.1128/msystems.01182-23)

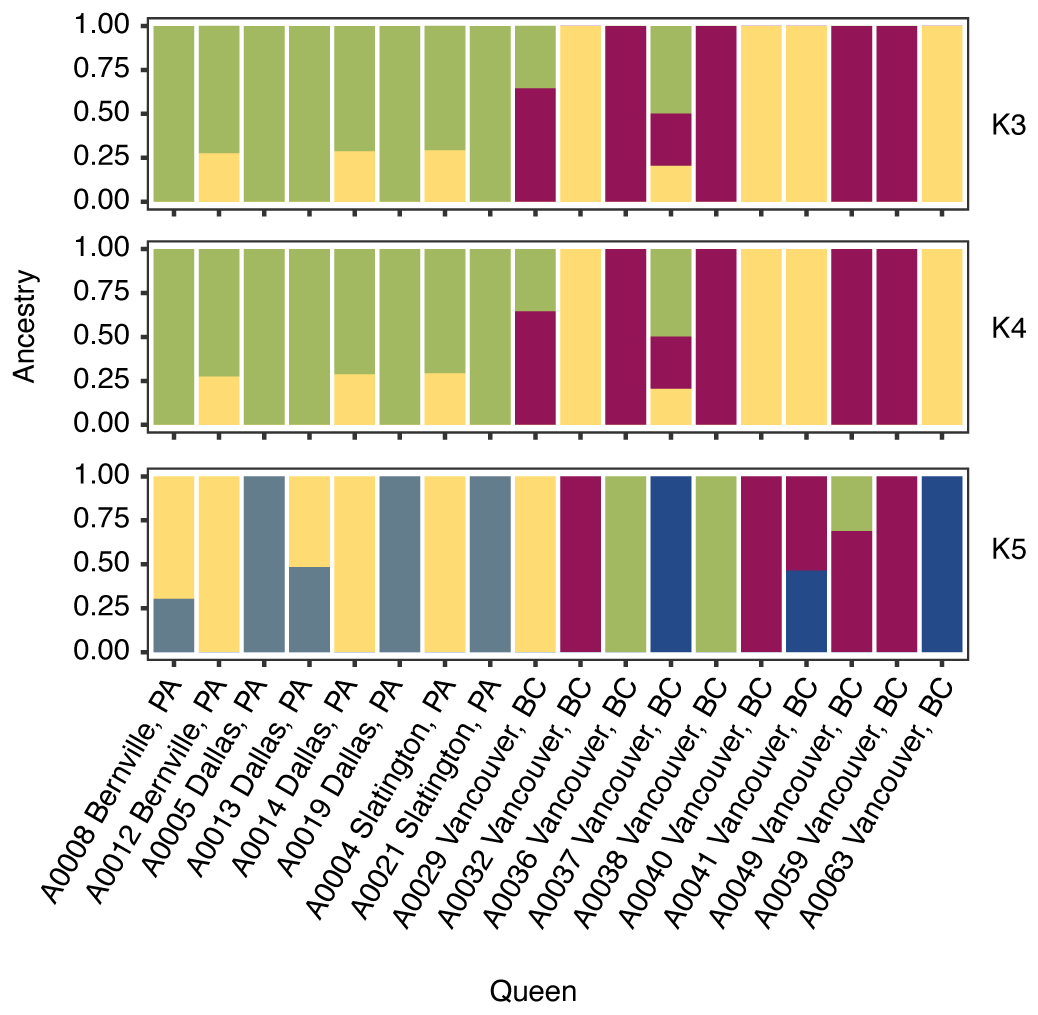

Supplement: Figure S1 — Admixture analysis of sampled queen populations. [file msystems.01182-23-s0002.tif]

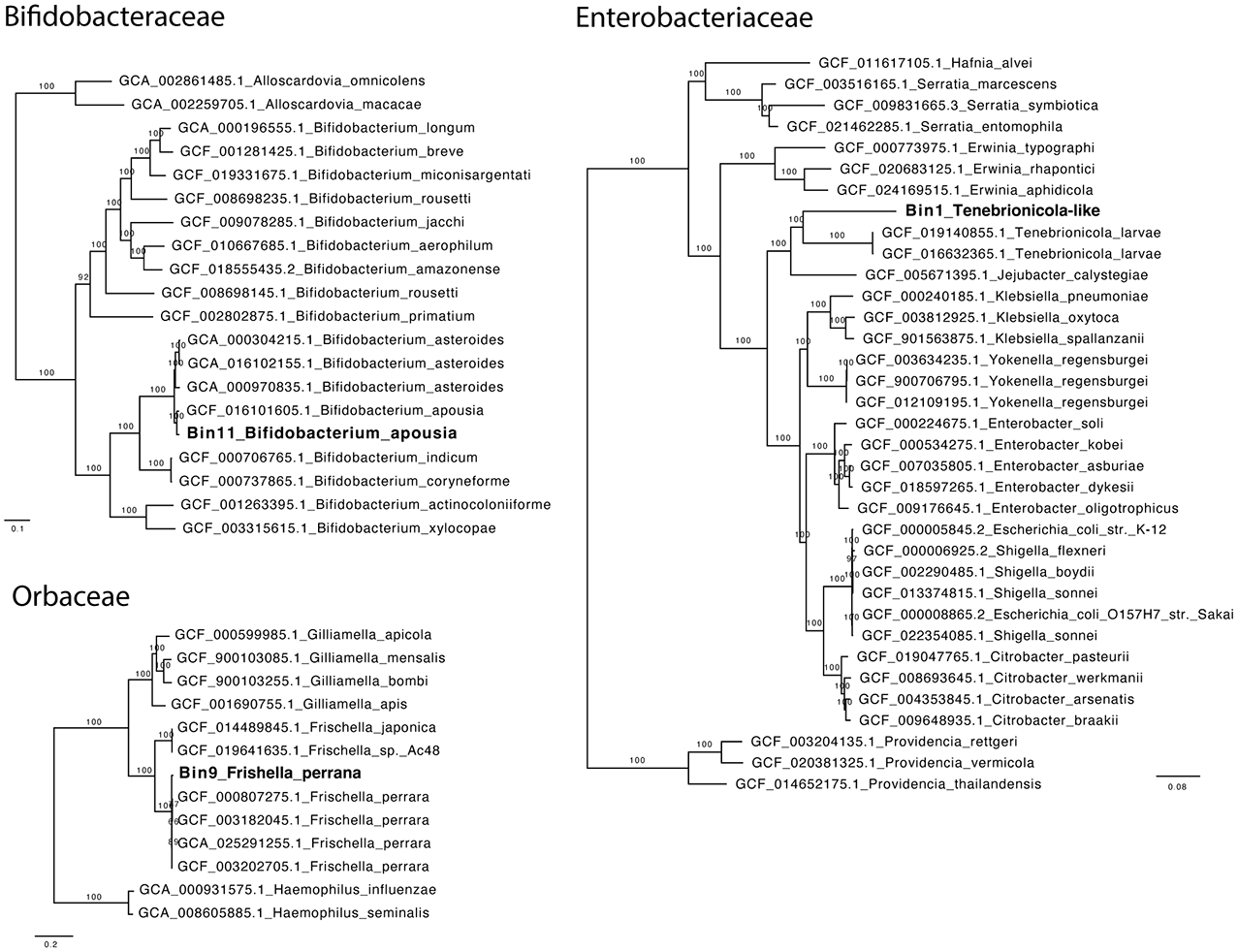

Supplement: Figure S2 — Phylogenies of non-core bacteria present in honey bee queens. [file msystems.01182-23-s0003.tif]

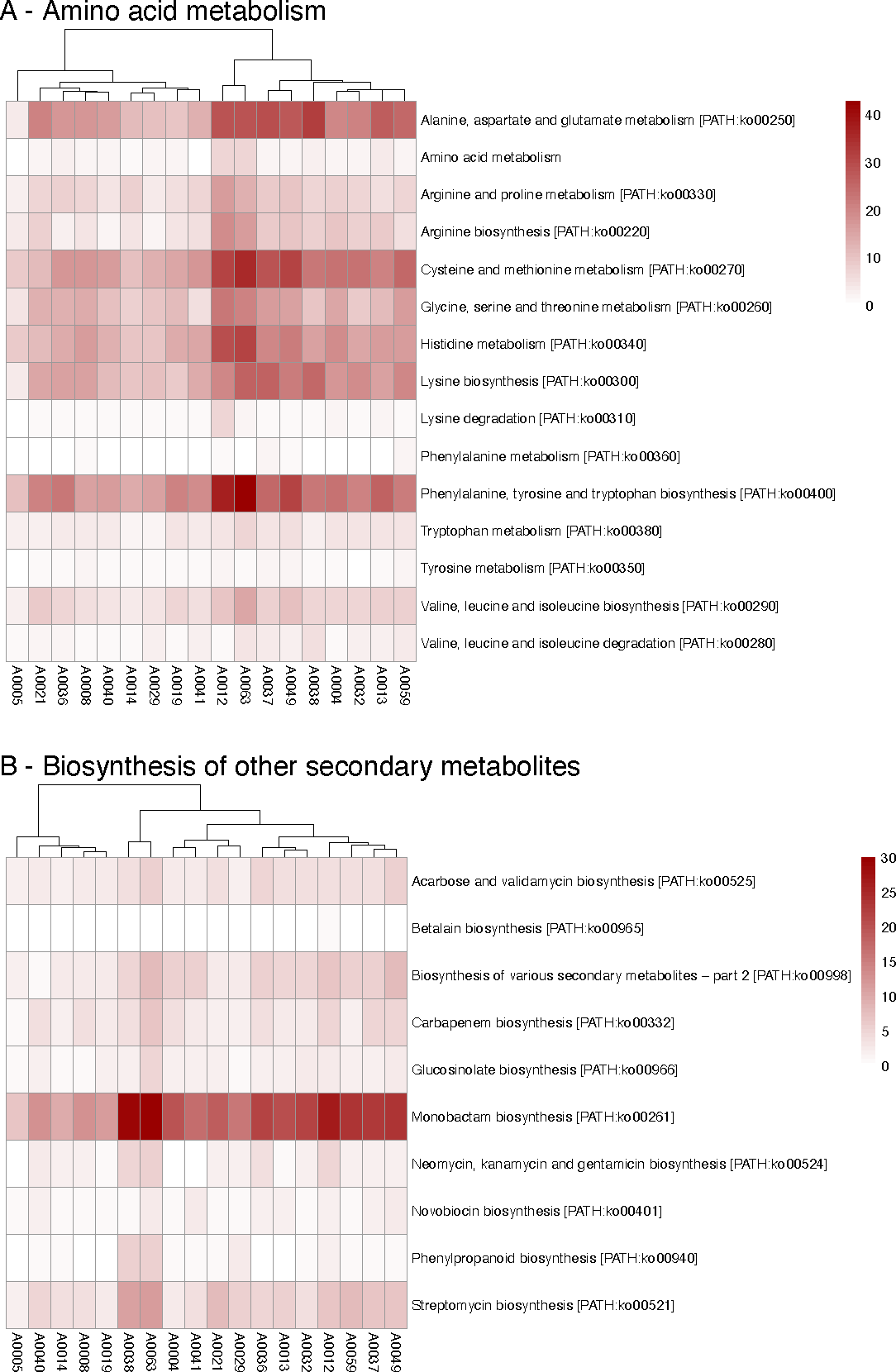

Supplement: Figure S3 — Zoom-in on non-general function KEGG categories. [file msystems.01182-23-s0004.tif]

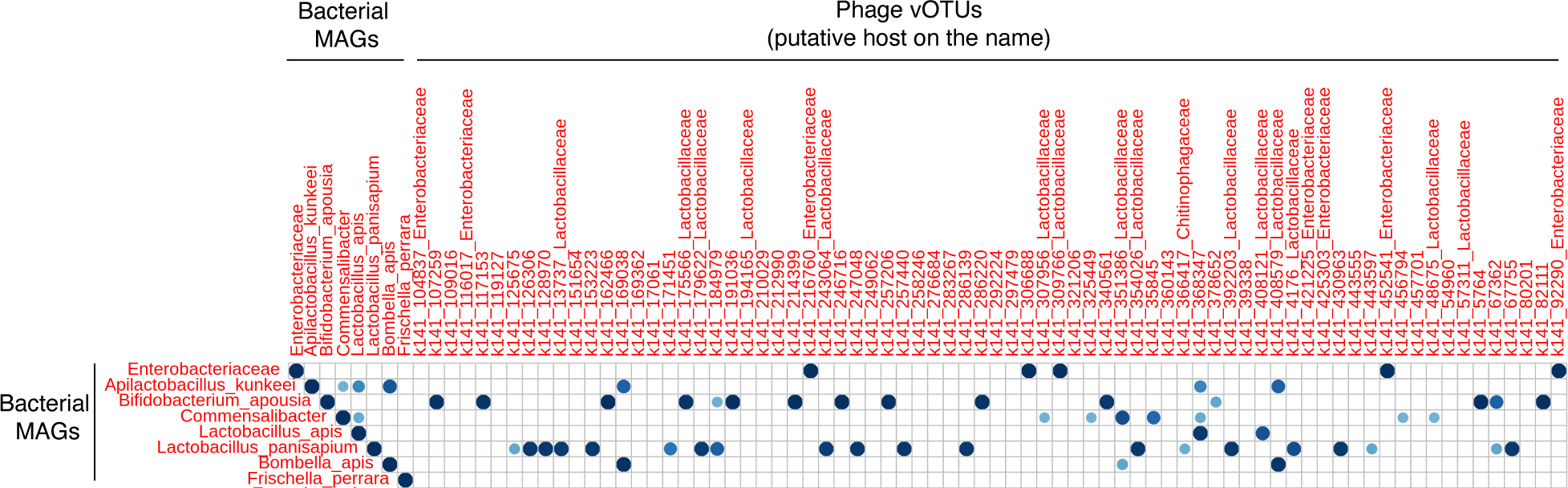

Supplement: Figure S4 — Correlation between MAG size and phage abundance. [file msystems.01182-23-s0005.tif]

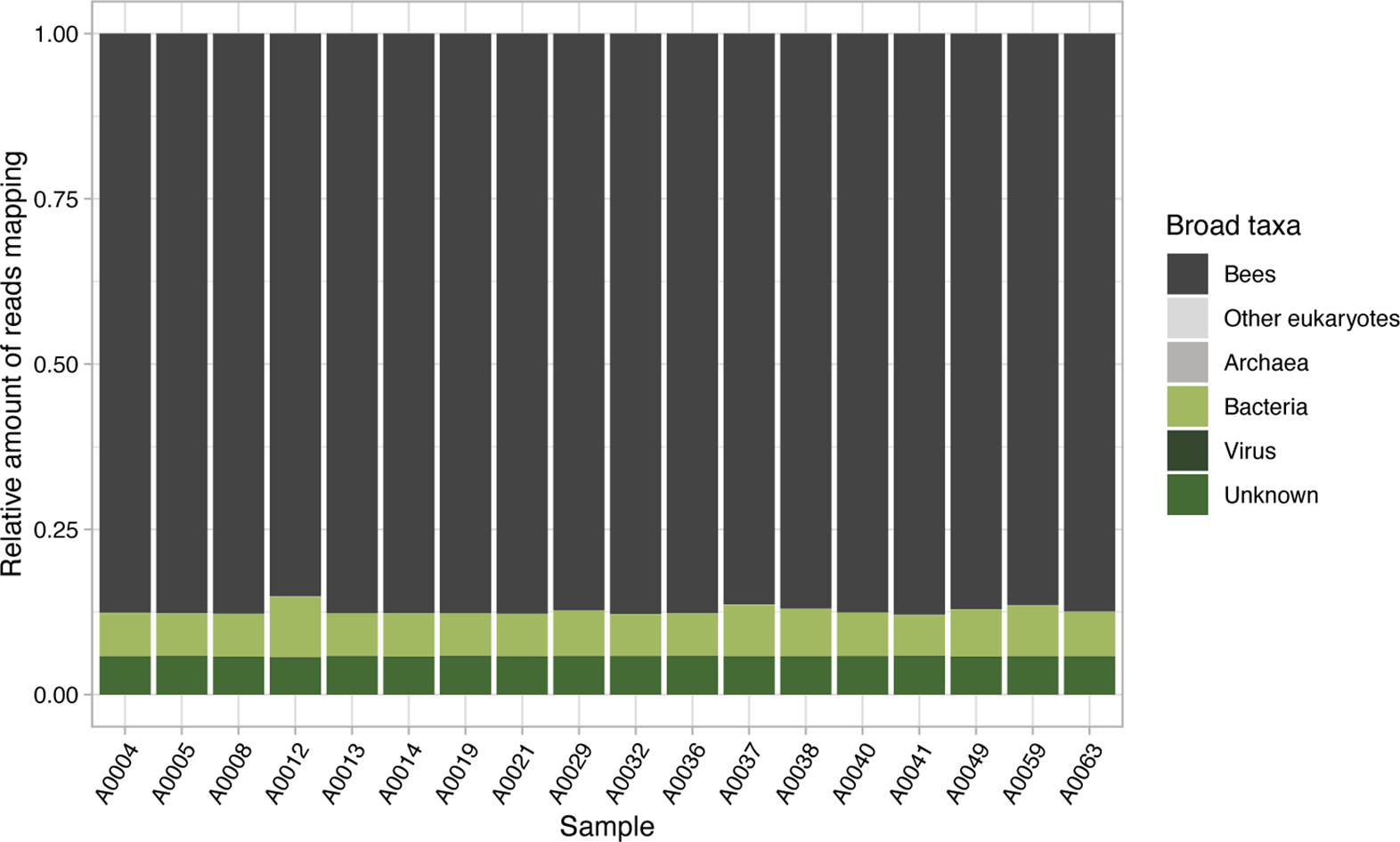

Supplement: Figure S5 — Proportion of reads mapping to host and microbes. [file msystems.01182-23-s0006.tif]
